# Prevalence and modifiable risk factors for dementia in persons with intellectual disabilities

**DOI:** 10.1186/s13195-023-01270-1

**Published:** 2023-07-18

**Authors:** Shintaro Takenoshita, Seishi Terada, Tomokazu Inoue, Taku Kurozumi, Norihito Yamada, Ryozo Kuwano, Shigeru Suemitsu

**Affiliations:** 1 Department of Neuropsychiatry, Okayama University Hospital, Okayama, Japan; 2 Department of Neuropsychiatry, Faculty of Medicine, Dentistry and Pharmaceutical Sciences, Okayama University, Okayama, Japan; 3 Asahigawaso Research Institute, Social Welfare Corporation Asahigawaso, 866 Gion, Kita-Ku, Okayama, 703-8555 Japan

**Keywords:** Cognitive impairment, Dementia, Intellectual disability, Prevalence of dementia, Risk factor

## Abstract

**Background:**

People with intellectual disability (ID) without Down syndrome (DS) are presumed to be at higher risk of developing dementia due to their lower baseline cognitive reserve. We aimed to determine the prevalence of dementia in people with ID without DS and to identify risk factors of dementia.

**Methods:**

This was a cross-sectional survey and multicenter study in Japan. Adults with ID without DS residing in the facilities were included. Caregivers of all participants were interviewed by medical specialists, and participants suspected of having cognitive decline were examined directly. ICD-10 criteria for dementia, DC-LD criteria for dementia, and DSM-5 criteria for neurocognitive disorders were used to diagnose dementia. The severity of ID, educational history, and comorbidities were compared by dividing the groups into those with and without dementia.

**Results:**

A total of 1831 participants were included; 118/1831 (6.44%) were diagnosed with dementia. The prevalence of dementia for each age group was 8.8%, 60–64 years; 9.0%, 65–69 years; 19.6%, 70–74 years; and 19.4%, 75–79 years. Age, severity of ID, duration of education, hypertension, depression, stroke, and traumatic brain injury were significantly associated with the presence of dementia.

**Conclusions:**

Although the prevalence of dementia in people with ID without DS was found to be higher at a younger age than in the general population, the results of this study suggested that adequate education, prevention of head trauma and stroke, and treatments of hypertension and depression may reduce the risk of dementia. These may be potentially important modifiable risk factors for the prevention of dementia in these people.

**Supplementary Information:**

The online version contains supplementary material available at 10.1186/s13195-023-01270-1.

## Background

Intellectual disability (ID) is a neurodevelopmental disorder resulting from multiple causes. Most cases of ID are due to genetic factors, but others are caused by acquired factors [1]. ID causes varying degrees of impairment in intelligence and adaptive function, its prevalence is estimated to be about 1%, and it is more common in males than females by about 20–30% [2]. In the past, the life expectancy of people with ID was much shorter than that of the general population due to the underdevelopment of medical management of physical complications such as cardiovascular, respiratory, and neoplastic diseases [3]. In recent years, the life expectancy of people with ID has gradually caught up with that of the general population [4]. As a result, dementia has become a bigger problem than ever before in communities where aging people with ID live [5]. Therefore, there is a need to understand the characteristics of dementia in people with ID and to identify information that can lead to prevention methods. Down syndrome (DS), 21 trisomy, accounts for about 9% of all ID [6]. DS is known to be affected by the amyloid precursor protein gene that is one of the causative genes for familial Alzheimer’s disease [7]. Individuals with DS develop dementia at a high frequency starting around age 40 [8].

On the other hand, the risk of dementia in people with ID without DS is not clear. It has been reported that a cognitive reserve, which is formed by education and cognitive stimulation, influences cognitive function in old age [9, 10]. Based on previous reports that low cognitive reserve in the general population increases the risk of dementia [9–12], it is speculated that the prevalence of dementia is higher in populations with ID, which tend to have less developed intellectual functioning and shorter duration of education [5, 13]. However, the results of previous reports are not consistent. Zigman et al. [14] reported that people with ID without DS were no more likely to develop dementia than the general population, while Strydom et al. [15] reported a higher prevalence and a shift in risk to younger age groups compared to the general population. These differences in study results can be attributed to differences in research methods (study population pools, criteria of ID, recruitment method, inclusion criteria, and method for diagnosis of dementia), but the lack of studies with a sufficient sample size of cases aged 65 + (126 cases for Zigman et al. [14] and 142 cases for Strydom et al. [15]) is another factor that may obscure conclusions. In addition, a relationship between duration of education and dementia in populations with ID has not been reported, and it is not clear whether education has the same effect on reducing the risk of dementia as it does in the general population. This study investigated the prevalence of dementia and dementia subtypes among people with ID living in institutions in a wide range of regions in Japan and examined the association between estimated risk factors for cognitive decline and dementia [16].

## Methods

### Ethics

The authors assert that all procedures contributing to this work comply with the ethical standards of the relevant national and institutional committees on human experimentation and with the Helsinki Declaration of 1975, as revised in 2008. All procedures involving human subjects/patients were approved by the ethical committee of the Okayama University and Asahigawaso Research Institute. It was registered at The University Hospital Medical Information Network Clinical Trials Registry (UMIN000038262). After giving a complete description of the study, written informed consent was obtained from the subjects who were judged to have the ability to express consent. In addition, written informed consent was obtained from the legal guardians including Legally Authorized Representative of all participants. This study followed the Strengthening the Reporting of Observational Studies in Epidemiology (STROBE) reporting guidelines for cross-sectional studies.

### Participants

Facility residents were included rather than home residents to avoid confounding factors due to differences in lifestyle. In 2019, there were 2149 residential facilities of various scales in Japan providing care for daily living for people with ID [17]. To avoid discrepancies among the investigators, surveys were conducted entirely by one team of investigators. The target time frame for completion of all surveys was 1 year. Therefore, to minimize the number of surveys required, and investigate a large number of cases efficiently, we limited our approach to large-scale social welfare corporations that are representative of their region. In Japan, social welfare corporations are nonprofit organizations established in accordance with the Social Welfare Law enacted in 1951. They provide social welfare services to support old people, people with disabilities or other vulnerable people, and are supervised by the Ministry of Health, Labor and Welfare. The Japanese archipelago is divided into three areas (northern, eastern, and western areas of Japan). To avoid sample bias, we asked the largest to the fourth largest social welfare corporation in each of the three areas. Nine social welfare corporations (with a total of 45 facilities) were willing to cooperate (one from northern, four from eastern, four from western Japan). Some regions (the Tohoku region in the northern region and the Kyushu region in the western region) were excluded from the survey because of natural disasters such as torrential rains and typhoons. The reasons why a  social welfare corporation refused to conduct the investigation were a small number of residents 20 years of age and older and difficulties in obtaining consent. Participants were recruited from the residents of these facilities in November 2019 according to the following inclusion criteria: (a) ID according to the criteria formulated by ICD-10 [18]: a condition of reduced overall level of intelligence (IQ < 70) that manifested during the developmental period; (b) information providers who had observed the living condition of the subject for 2 years or more were available; (c) the subject was 20 years or older; and (d) the absence of DS could be verified by the facility’s records. There were no differences in the criteria for admission to the facilities of each corporation.

### Information collection and diagnosis process

#### Step 1: Informants responded to the survey form

A caregiver for each participant who had been involved with him or her for more than 2 years and who knew the changes in the functions of the participant was designated as the informant. The informant completed a detailed survey form on baseline intellectual functioning, educational history, medical history, basic activities of daily living (e.g., toileting, feeding, dressing, grooming, ambulation, and bathing), and instrumental activities of daily living (e.g., shopping, telephoning, and laundry) by reviewing clinical records maintained by the facility [19]. The survey form included the Japanese version of the Dementia Screening Questionnaire for Individuals with Intellectual Disabilities (DSQIID-J), which is an observer-rating dementia screening tool for people with ID [20, 21]. The maximum score was 53 (cut-off score 10/11, sensitivity 100%, specificity 96.8%). The higher the DSQIID-J score, the greater the decline in cognitive function over time.

#### Step 2: Investigating physicians interviewed informants

Two research physicians (S.Ta. and R.K.) interviewed each informant to confirm the status of the participant, his/her past progress, and information on the survey form. The participants who were judged by the physicians to have a possibility of progression of cognitive impairment or decline in activities of daily living based on information obtained and participants with a DSQIID-J score of 11 or more points were identified as possible cases of dementia or mild cognitive impairment (MCI).

#### Step 3: Investigating physicians examined each participant

Two research physicians (S.Ta. and R.K.) directly examined the participants who were identified as possible cases of dementia or MCI. The examination of each participant reviewed the function in each neurocognitive domain (complex attention, executive function, learning and memory, language, perception and movement, and social cognition), neurological symptoms related to dementia, and status of physical comorbidities which were assessed and recorded [1]. The severity of ID was determined based on ICD-10 Research Diagnostic Criteria [18].

#### Step 4: Participants were diagnosed

Three research physicians (S.Ta., R.K., S.Te.) diagnosed dementia and MCI based on each diagnostic criterion, referring to all information gathered up to Step 3. Any disagreement was resolved by discussion among the three. The diagnostic process took into account each case’s baseline level of intellectual and adaptive function, comorbidity status and course, the effects of medication, age-related sensory and motor function loss, and influences of environmental change or life events. Because baseline functions of people with ID vary from individual to individual, the decline of cognitive function or performance of activities of daily living was judged significant when a clear loss of function from the individual’s previous level of functioning, rather than deviation from the normal level of the general population. In cases of having comorbid mental illnesses such as autism spectrum disorders and depression, dementia was diagnosed when the functional decline could not be explained by the mental illness. The final judgment was made according to the wording of each diagnostic criteria.

### Diagnostic criteria

In diagnosing dementia, we used three criteria: ICD-10 Research Diagnostic Criteria for dementia [18], Diagnostic Criteria for Psychiatric Disorders for Use with Adults with Learning Disabilities/Mental Retardation (DC-LD) [22] criteria for dementia, and Diagnostic and Statistical Manual of Mental Disorders, 5th Edition (DSM-5) [1] criteria for neurocognitive disorders. We used the National Institute on Aging-Alzheimer’s Association workgroups criteria for Alzheimer’s disease dementia (AD) [23], the American Heart Association/American Stroke Association criteria for vascular dementia (VaD) [24], the 2017 Consortium on Dementia with Lewy Bodies criteria for dementia with Lewy bodies (DLB) [25], and the International Consensus Criteria for Behavioural Variant FTD for behavioral variant frontotemporal dementia (bvFTD) [26].

MCI is defined as a condition that is intermediate between normal cognition and dementia. The methods for diagnosing MCI in the general population are not suitable for people with ID, who have pre-existing cognitive impairment, and there are no clear diagnostic criteria applicable to this population [5]. By modifying the criteria of Petersen et al. (1999, 2011) [27, 28] for adults with ID, this study defined MCI as a condition that meets all of the following criteria: (1) cognitive function is decreased compared to the past; (2) decreased function in one or more of the following cognitive domains: memory, executive function, attention, language, and visuospatial cognition; (3) activities of daily living are not clearly lost, although efficiency may be less than before, mistakes may be increased, and more help from caregivers may be required; and (4) is not dementia. DSM-5 criteria were used to diagnose MCI subtypes [1].

### Statistical analysis

Statistical analysis was performed using the SPSS 24.0 J software program (SPSS Inc., Chicago, Ill., USA). Comparisons between groups were computed using Student’s *t*-test for continuous data. Categorical variables were analyzed by Pearson’s χ^2^ test. Binary logistic regression analysis was performed to estimate the risk of dementia with potential predictors, including demographic variables (age, sex, and length of education) and clinical variables such as severity of ID, vision disorder, hearing disorder, gait disorder, hypertension, dyslipidemias, diabetes, depression, traumatic brain injury (TBI), and stroke. The variables included in the analysis were factors based on existing knowledge of risk factors for dementia and factors whose presence or absence could be properly assessed and presumed to be potential risk factors. Factors with *P* > 0.2 in the two-group comparison were excluded. Spearman’s rank correlation test was used to check for multicollinearity. All reported *P* values were 2-tailed, and significance was set at* P* < 0.05.

## Results

### Demographics

A total of 1831 adults with ID without DS were included in the study. There were 1969 adults with ID at facilities who were potential participants; 133 (6.75%) with DS and 5 who died or left the facility during the study period were excluded. Consent to participate in this study was obtained from all participants. The survey was conducted between October 2019 and November 2020, and the data were analyzed between March 2021 and June 2021. The demographic details are shown in Table 1. Of the 1831 participants (age range, 20–97 years; mean, 54.7 years; SD: 12.4), 118/1831 (6.4%) were diagnosed with dementia (mean, 71.7 years; SD: 10.1) and 50/1831 (2.7%) with MCI (mean, 70.0 years; SD: 7.6) The prevalence of dementia for each age group was 8.8%, 60–64 years; 9.0%, 65–69 years; 19.6%, 70–74 years; 19.4%, 75–79 years; 26.2%, 80–84 years; and 27.8%, 85–90 years. Demographic details by age group about diagnosis for dementia and MCI are shown in Supplementary table 1, and details in each corporation are shown in Supplementary table 2.

**Table 1:** Demographic details of participants

Demographics	Total (*n* = 1831)	Dementia (*n* = 118)	Without dementia (*n* = 1713)	*P-*value
Age, mean years (SD)	54.7 (12.4)	71.7 (10.1)	53.6 (14.5)	** < 0.001**
Female sex, *n* (%)	712 (38.9)	62 (52.5)	650 (37.9)	**0.002**
Male sex, *n* (%)	1119 (61.1)	56 (47.5)	1063 (62.1)	
Education, mean years (SD)^a^	8.3 (3.2)	4.4 (4.6)	8.5 (4.0)	** < 0.001**
Severity of ID				
Mild ID, *n* (%)	50 (2.7)	5 (4.2)	45 (2.6)	0.45
Moderate ID, *n* (%)	379 (20.7)	21 (17.8)	358 (20.9)	
Severe ID, *n* (%)	1402 (76.6)	92 (78.0)	1310 (76.5)	
DSQIID-J, mean (SD)	3.4 (6.5)	17.8 (12.9)	2.4 (4.3)	** < 0.001**
Observation period, mean years (SD)	7.9 (6.8)	10.0 (7.5)	7.8 (6.7)	**0.002**
Comorbidity				
Vision disorder, *n* (%)	122 (6.7)	15 (12.7)	107 (6.2)	**0.006**
Hearing disorder,* n* (%)	106 (5.8)	17 (14.4)	89 (5.2)	** < 0.001**
Gait disorder,* n* (%)	33 (1.8)	4 (3.4)	29 (16.9)	0.18
Hypertension,* n* (%)	288 (12.5)	53 (45.0)	235 (13.7)	** < 0.001**
Dyslipidemias,* n* (%)	219 (12.0)	25 (21.2)	194 (11.3)	**0.001**
Diabetes,* n* (%)	96 (5.2)	10 (8.5)	86 (5.0)	0.10
Depression,* n* (%)	29 (1.6)	4 (3.4)	25 (1.5)	0.11
TBI,* n* (%)	40 (2.2)	10 (8.5)	30 (1.8)	** < 0.001**
Stroke,* n* (%)	52 (2.8)	22 (18.6)	30 (17.5)	** < 0.001**
Epilepsy,* n* (%)	782 (42.7)	45 (38.1)	737 (43.0)	0.30

*Abbreviations: DSQIID-J* The Japanese version of the Dementia Screening Questionnaire for Individuals with Intellectual Disabilities, *ID* intellectual disabilities, *SD* standard deviation, *TBI* traumatic brain injury

*P* value compares subjects with and without dementia

^a^For 1682/1831 (91.8%), the duration of education was confirmed

**Fig. 1:**
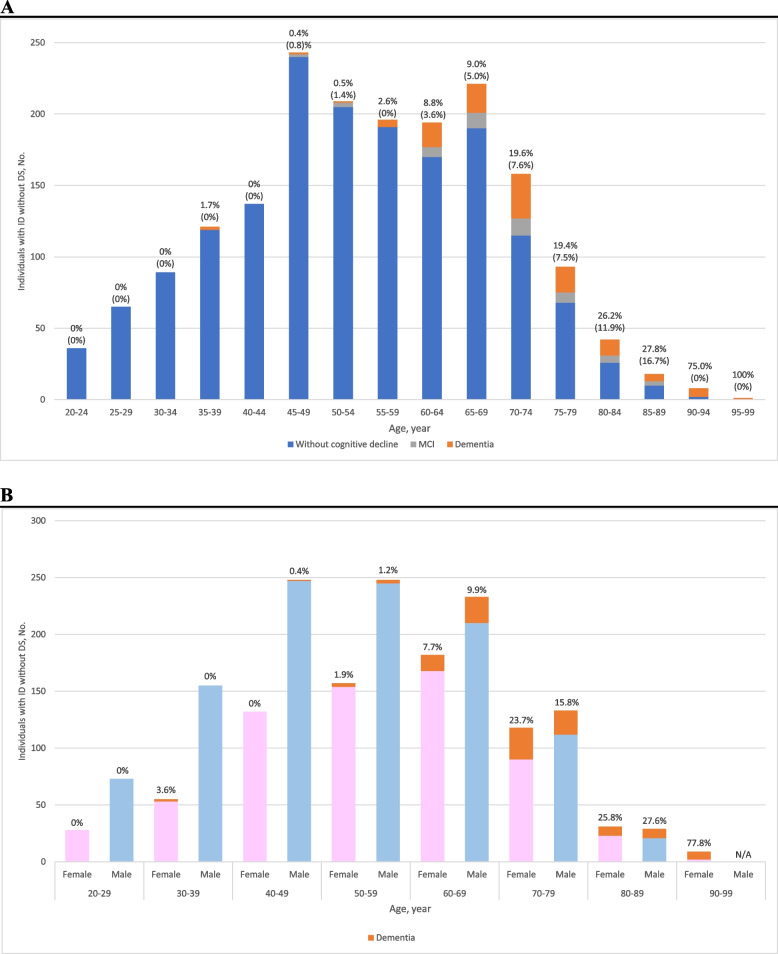
**A** Histogram and prevalence of dementia and MCI. **B** Prevalence of dementia by age: male and female. Abbreviation: MCI, mild cognitive impairment. The prevalence of dementia and MCI are shown above each bar graph, and the prevalence of MCI is shown in parentheses

All 118 dementia patients met the DSM-5 criteria (details of meeting each diagnostic criterion are shown in Supplementary table 3). Compared to the non-dementia group, the dementia group was older and had a shorter education duration. Histograms and age-specific prevalence of dementia and MCI are shown in Fig. 1. The number of cases diagnosed with dementia increased after the age of 55 as the population aged. There was no clear difference between males and females in the prevalence of dementia by age.

### Subtypes of dementia and MCI

**Fig. 2:**
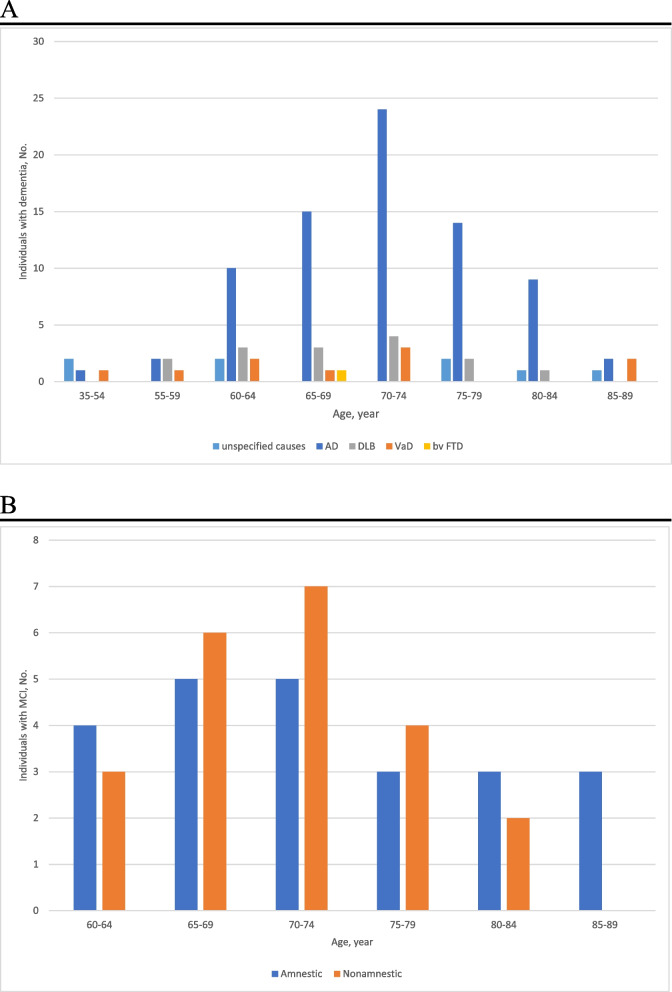
**A** Subtypes of dementia by age group. **B** Subtypes of MCI by age group. Abbreviations: AD, Alzheimer’s disease dementia; VaD, vascular dementia; DLB, dementia with Lewy bodies; bvFTD, behavioral variant FTD; MCI, mild cognitive impairment

Of the 118 patients with dementia, 82/118 (69.5%) were classified as having probable AD, 14/118 (11.9%) as probable DLB, 13/118 (11.0%) as probable VaD, 1/118 (0.8%) as possible bvFTD, and 8/118 (6.8%) as dementia due to unspecified causes (Fig. 2). Of the 50 patients with MCI, 27/50 (54%) had amnestic MCI and 23/50 (46%) had non-amnestic MCI. Of the presumed background diseases, 34/50 (68%) were classified as due to AD, 6/50 (12%) as due to DLB, 1/50 (2%) as due to VaD, and 9/50 (18%) as unspecified.

### Years of education and dementia

The duration of education was confirmed in 1682 (91.8%) of 1831 subjects. The dementia group had significantly fewer years of education than the non-dementia group (mean, 4.4 years; SD: 4.6 *vs*. mean, 8.5 years; SD: 4.0).

### Comorbidity and dementia

Comparison of the dementia and non-dementia groups by comorbidities are shown in Table 1. As described in the “Methods” section, the factors of estimated risk for dementia were examined by logistic regression analysis, and significant differences were found in the following factors (Table 2): age (OR 1.10, 95% CI 1.07–1.12, *P* < 0.001), years of education (OR 0.94, 95% CI 0.89–0.99, *P* = 0.024), severity of ID (OR 1.93, 95% CI 1.08–3.43, *P* = 0.025), hypertension (OR 2.11, 95% CI 1.31–3.41,* P* = 0.002), depression (OR 4.28, 95% CI 1.28–14.29, *P* = 0.018), TBI (OR 5.03, 95% CI 2.08–12.19, *P* < 0.001), and stroke (OR 10.94, 95% CI 4.50–26.58, *P* < 0.001). Spearman’s rank correlation test was employed to check for multicollinearity. No variable showed a high association with Spearman coefficient | *r* |> 0.70. The prevalence of each comorbidity by age group is shown in Supplementary table 4.

**Table 2:** Prediction models for the probability of dementia in people with intellectual disability

	Odds ratio (95% CI)	*P*-value	Beta coefficient
Age, per year	1.10 (1.07–1.12)	** < 0.001**	0.093
Sex, female *v*. male	1.04 (0.66–1.64)	0.861	0.041
Years of education, per year	0.94 (0.89–0.99)	**0.024**	− 0.064
Severity of ID, mild or moderate *vs*. severe	1.93 (1.08–3.43)	**0.025**	0.657
Comorbidity			
Vision disorder	1.67 (0.83–3.34)	0.148	0.512
Hearing disorder	1.56 (0.78–3.10)	0.205	0.444
Gait disorder	0.84 (0.21–3.34)	0.805	-0.174
Hypertension	2.11 (1.31–3.41)	**0.002**	0.748
Dyslipidemias	0.80 (0.44–1.45)	0.461	-0.222
Diabetes	0.80 (0.35–1.84)	0.601	-0.221
Depression	4.28 (1.28–14.29)	**0.018**	1.453
TBI	5.03 (2.08–12.19)	** < 0.001**	1.616
Stroke	10.94 (4.50–26.58)	** < 0.001**	2.392

*Abbreviations: ID* intellectual disabilities; *TBI* traumatic brain injury

## Discussion

### Prevalence of dementia with ID without DS

The difference between the prevalence of dementia in the general population in Japan and this study is shown in Table 3 [29]. The results of this study show that the prevalence of dementia among people with ID without DS is clearly higher in the age group between 65 and 80 years old than in the general population. However, in the age group between 85 and 90 years old, the prevalence of dementia was higher in the general population than in the people with ID. Although the sample size of this study aged 80 + was smaller than that of other age groups and is not sufficient to be reliable, there are two possible reasons for the reversal of prevalence rates between the people with ID and the general population. First, this study may not include all dementia cases because some cases move to hospitals or nursing homes for the elderly as their physical functions decline with age. Second, because people with ID tend to have shorter life spans than the general population, long-lived adults with ID who survive beyond age 85 may be a rare “healthy population.”

**Table 3:** Prevalence of dementia in people with ID compared to the general population in Japan

This study (people with ID without DS in Japan)			Ninomiya et al. [29] (General population in Japan)	
**Total subjects, *n***	**Dementia, *n* (%)**	**Age, years**	**Total subjects, *n***	**Dementia, n (%)**
194	17 (8.8)	60–64	NA	NA
221	20 (9.0)	65–69	3728	33 (0.9)
158	31 (19.6)	70–74	2758	65 (2.4)
93	18 (19.4)	75–79	2218	114 (5.1)
42	11 (26.2)	80–84	1462	231 (15.8)
18	5 (27.8)	85–89	816	279 (34.2)
9	7 (77.8)	90 +	428	245 (57.2)

*Abbreviations: DS* Down syndrome, *ID* intellectual disabilities, *NA* not applicable

Reference: [29]

The next question of interest is whether there is a difference in the prevalence of dementia between people with ID living in facilities and out of facilities. In our previous cross-sectional study of people with ID without DS in Japan (*n* = 459), 39.7% of participants were living out of a facility (independent or group home) [30]. The prevalence of dementia in the previous study including non-institutionalized cases, and the present study including only institutionalized cases was generally identical when age groups with a sufficient population (1/134, 0.8% vs. 2/452, 0.4%, 45–54 years; 3/86, 3.5% *vs*. 22/390, 5.6%, 55–64 years; 5/36, 13.9% *vs*. 51/379, 13.4%, 65–74 years) were compared [30].

### Effects of pre-existing ID and education on dementia risk

Many previous studies have consistently documented that individuals who are highly educated or frequently participate in intellectual activity may have better cognitive functioning in old age and a lower risk of developing dementia [9–12]. On the other hand, one negative effect that may affect the risk of dementia in people with ID is the wear-and-tear phenomenon (proposed under the name of the “weathering hypothesis”), whereby social minorities are subjected to so much stress from socioeconomic disadvantage that they carry an allostatic load, which accelerates biological aging and causes cognitive decline [31, 32]. However, there have been no studies that clearly show how the severity of pre-existing ID and educational history are related to the risk of dementia in people with ID [33]. Strydom et al. [34] stated that the prevalence of dementia does not vary with the severity of ID. In this study, we conducted a logistic regression analysis including the severity of ID, years of education, and comorbidities. The results showed that more severe ID was associated with an increased risk of dementia, and a longer educational period was associated with a reduced risk of dementia. Although there have been previous reports on cognitively healthy adults that the development of verbal intelligence has a greater effect on cognitive reserve than the duration of education [35], the current study shows that adequate education may ameliorate the development of dementia, even when preexisting ID is severe. In Japan, all people with ID including profound ID are given a special education of at least 9 years including learning support and daily life training with a curriculum tailored to their individual abilities. However, since the uniform planned education system in all regions began in 1979, it is assumed that those who received education before that time (persons 46 years of age or older in this study) had regional differences in the educational curriculums provided [36]. It is important to note that the educational content for people with ID varies by region and time.

### Comorbidity as a risk of dementia

Reports in the general population have associated hypertension, diabetes, obesity, hearing disorder, TBI, depression, smoking, physical inactivity, and social isolation with increased risk of dementia [16]. The lifestyles of people with ID are diverse, so it is necessary to pay attention to confounding factors when discussing the relationship between ID itself and dementia. On the other hand, the subjects of this study are all facility residents, so they are a homogeneous group with a uniform lifestyle. They are living in a group receiving nutritional care, support for physical exercise, and encouragement for intellectual activities, while smoking is prohibited. In the analysis, hypertension, dyslipidemias, diabetes, depression, TBI, stroke, hearing disorder, vision disorder, and gait disorder were included among the comorbidities in the logistic regression analysis. Among the comorbidities, hypertension, depression, TBI, and stroke were associated with the risk of dementia. The prevalence of TBI in this study was surprisingly higher than that in the general population (2.2% *vs*. 0.5%) [37]. These results suggest that adequate head protection is important to prevent dementia in people with ID. The prevalence of hypertension in the study was lower than that in the general population (15.7% *vs*. 22.5%) [38]. Even when living in a nutritionally controlled environment to avoid excessive salt intake, hypertension is still likely to be a high-risk factor for dementia, suggesting the need for constant attention to the management of hypertension. Although diabetes and hearing disorders are clearly identified risks for dementia in the general population, they did not show a significant association with dementia in this study. The prevalence of diabetes in this study and that in the general population was almost the same (5.2% and 5.6%) [39]. Facility residents are protected from aggravating factors such as alcohol consumption and smoking, which worsen insulin resistance, so differences in living environment may explain why diabetes is a risk factor for dementia in the general population but not in this study. Although hearing disorders are also an identified risk for dementia in the general population, logistic regression analysis in this study did not show a significant association with dementia. The prevalence of hearing disorders in this study was 10.9% in the age group 65–69 years (Supplementary Table 4), while in the general Japanese population it is 43.7% in the same age group [40]. The large difference in the prevalence of hearing disorders suggests that people with ID may not be adequately evaluated for age-related hearing disorder. In addition, hearing and vision impairments may already be included in the severity of ID, which may also be a reason why no association with dementia was found.

### Limitations

There are some limitations in this study. First, the sample size of those aged 80 + is relatively small (69 participants). Thus, we may underestimate the prevalence of dementia in the group older than 80. We do not know the specific number of cases leaving facilities before death due to aging and increasing medical needs. Second, the presence or absence of comorbidities (e.g., hypertension and depression) and risk factors is based on personal information registered at the facilities and was not reassessed at the time this study was conducted. Therefore, they may deviate from the exact real numbers. Third, the design of this study was a cross-sectional study, which limits the ability to conclude causality, whether it is a risk factor or a symptom resulting from dementia.

### Implications

This study has an adequate sample size of cases aged 65 to 80 years old to discuss the prevalence of dementia. To our knowledge, this is the largest study of the prevalence of dementia in the population with ID to date. This study obviated inter-rater discrepancies by having all participants examined and diagnosed by the same research physicians in a consistent manner. The problem of selection bias was also solved by the fact that this study includes participants from various geographic regions throughout Japan and by the high study participation rate achieved. This study found that the prevalence of dementia in people with ID without DS was higher at younger ages than in the general population. This is the first study to show that there may be several effective interventions to minimize the development of dementia in people with ID. Although a higher baseline severity of ID may increase the risk of dementia, the provision of adequate education, prevention of head trauma and stroke, and treatments of hypertension and depression may reduce the risk of dementia in people with ID. The control of potentially modifiable risk factors, based on the evidence of this study, may be the key to long-term care in the people with ID as high-risk group of dementia.

## Supplementary Information

**Additional file 1:** **Supplementary table 1.** Demographic details of cognitive decline by age group.

**Additional file 2:** **Supplementary table 2.** Demographic details in each corporation by age group.

**Additional file 3:** **Supplementary table 3.** Diagnosis of dementia differences by diagnostic criteria.

**Additional file 4:** **Supplementary table 4.** Prevalence of comorbidities by age group and demographic details.

## Data Availability

The datasets used and/or analyzed during the current study are available from the corresponding author on reasonable request.

## Abbreviations

ID: Intellectual disability
DS: Down syndrome
DSQIID-J: The Japanese version of the Dementia Screening Questionnaire for Individuals with Intellectual Disabilities
MCI: Mild cognitive impairment
DC-LD: Diagnostic Criteria for Psychiatric Disorders for Use with Adults with Learning Disabilities/Mental Retardation
DSM-5: Diagnostic and Statistical Manual of Mental Disorders, 5th Edition
AD: Alzheimer’s disease dementia
VaD: Vascular dementia
DLB: Dementia with Lewy bodies
bvFTD: Behavioral variant frontotemporal dementia
TBI: Traumatic brain injury
