# Supplementary material for: Prevalence and modifiable risk factors for dementia in persons with intellectual disabilities
Source: Alzheimers Res Ther. 2023 Jul 18;15:125. doi: 10.1186/s13195-023-01270-1 (PMC10354971; doi:10.1186/s13195-023-01270-1)
Supplement: Supplementary file 1 — Additional file 1: Supplementary table 1. Demographic details of cognitive decline by age group. [file 13195_2023_1270_MOESM1_ESM.docx]

**Supplementary table 1. Demographic details of cognitive decline by age group**

| **Age, years** | **Total, *n***  **(*n* = 1831)** | **Dementia, *n***  **(*n* = 118)** | **MCI, *n***  **(*n* = 50)** |
| --- | --- | --- | --- |
| 20-24 | 36 | 0 | 0 |
| 25-29 | 65 | 0 | 0 |
| 30-34 | 89 | 0 | 0 |
| 35-39 | 121 | 2 | 0 |
| 40-44 | 137 | 0 | 0 |
| 45-49 | 243 | 1 | 2 |
| 50-54 | 209 | 1 | 3 |
| 55-59 | 196 | 5 | 0 |
| 60-64 | 194 | 17 | 7 |
| 65-69 | 221 | 20 | 11 |
| 70-74 | 158 | 31 | 12 |
| 75-79 | 93 | 18 | 7 |
| 80-84 | 42 | 11 | 5 |
| 85-89 | 18 | 5 | 3 |
| 90-94 | 8 | 6 | 0 |
| 95-99 | 1 | 1 | 0 |

Abbreviation: MCI, Mild cognitive impairment.
