# Supplementary material for: Prevalence and modifiable risk factors for dementia in persons with intellectual disabilities
Source: Alzheimers Res Ther. 2023 Jul 18;15:125. doi: 10.1186/s13195-023-01270-1 (PMC10354971; doi:10.1186/s13195-023-01270-1)
Supplement: Supplementary file 2 — Additional file 2: Supplementary table 2. Demographic details in each corporation by age group. [file 13195_2023_1270_MOESM2_ESM.docx]

**Supplementary table 2. Demographic details in each corporation by age group**

| **Age, years** | **Corporation 1** | | **Corporation 2** | | **Corporation 3** | |
| --- | --- | --- | --- | --- | --- | --- |
|  | **Total, *n*** | **Dementia, *n*** | **Total, *n*** | **Dementia, *n*** | **Total, *n*** | **Dementia, *n*** |
| 20-29 | 11 | 0 | 24 | 0 | 8 | 0 |
| 30-39 | 33 | 0 | 47 | 1 | 14 | 0 |
| 40-49 | 56 | 0 | 92 | 0 | 32 | 0 |
| 50-59 | 64 | 0 | 78 | 1 | 18 | 0 |
| 60-69 | 53 | 3 | 80 | 6 | 18 | 0 |
| 70-79 | 7 | 0 | 21 | 5 | 34 | 8 |
| 80-89 | 2 | 0 | 0 | 0 | 7 | 2 |
| 90-99 | 0 | 0 | 0 | 0 | 0 | 0 |

| **Age, years** | **Corporation 4** | | **Corporation 5** | | **Corporation 6** | |
| --- | --- | --- | --- | --- | --- | --- |
|  | **Total, *n*** | **Dementia, *n*** | **Total, *n*** | **Dementia, *n*** | **Total, *n*** | **Dementia, *n*** |
| 20-29 | 22 | 0 | 4 | 0 | 21 | 0 |
| 30-39 | 41 | 1 | 11 | 0 | 25 | 0 |
| 40-49 | 67 | 0 | 18 | 0 | 38 | 0 |
| 50-59 | 76 | 1 | 9 | 0 | 77 | 0 |
| 60-69 | 58 | 6 | 10 | 0 | 76 | 4 |
| 70-79 | 32 | 6 | 6 | 1 | 60 | 8 |
| 80-89 | 5 | 1 | 2 | 0 | 13 | 3 |
| 90-99 | 0 | 0 | 0 | 0 | 4 | 4 |

| **Age, years** | **Corporation 7** | | **Corporation 8** | | **Corporation 9** | |
| --- | --- | --- | --- | --- | --- | --- |
|  | **Total, *n*** | **Dementia, *n*** | **Total, *n*** | **Dementia, *n*** | **Total, *n*** | **Dementia, *n*** |
| 20-29 | 4 | 0 | 0 | 0 | 7 | 0 |
| 30-39 | 17 | 0 | 0 | 0 | 22 | 0 |
| 40-49 | 17 | 0 | 10 | 0 | 50 | 1 |
| 50-59 | 9 | 0 | 26 | 2 | 48 | 2 |
| 60-69 | 5 | 0 | 66 | 14 | 49 | 4 |
| 70-79 | 3 | 1 | 60 | 13 | 28 | 7 |
| 80-89 | 0 | 0 | 12 | 2 | 19 | 8 |
| 90-99 | 0 | 0 | 2 | 2 | 3 | 1 |
