# Supplementary material for: Prevalence and modifiable risk factors for dementia in persons with intellectual disabilities
Source: Alzheimers Res Ther. 2023 Jul 18;15:125. doi: 10.1186/s13195-023-01270-1 (PMC10354971; doi:10.1186/s13195-023-01270-1)
Supplement: Supplementary file 3 — Additional file 3: Supplementary table 3. Diagnosis of dementia differences by diagnostic criteria. [file 13195_2023_1270_MOESM3_ESM.docx]

**Supplementary table 3. Diagnosis of dementia differences by diagnostic criteria**

| **DSM-5** | **ICD-10** | **DC-LD** | **Number of cases with dementia, *n*** |
| --- | --- | --- | --- |
| + | + | + | 87 |
| + | - | - | 29 |
| + | + | - | 1 |
| + | - | + | 1 |

Abbreviations: DSM-5, Diagnostic and Statistical Manual of Mental Disorders, 5th Edition; ICD-10, ICD-10 Research Diagnostic Criteria for dementia; DC-LD, Diagnostic Criteria for Psychiatric Disorders for Use with Adults with Learning Disabilities/Mental Retardation.

The case that meets each diagnostic criterion is indicated as +, and the case that does not meet the criterion is indicated as -.
