# Supplementary material for: Prevalence and modifiable risk factors for dementia in persons with intellectual disabilities
Source: Alzheimers Res Ther. 2023 Jul 18;15:125. doi: 10.1186/s13195-023-01270-1 (PMC10354971; doi:10.1186/s13195-023-01270-1)
Supplement: Supplementary file 4 — Additional file 4: Supplementary table 4. Prevalence of comorbidities by age group and demographic details. [file 13195_2023_1270_MOESM4_ESM.docx]

**Supplementary table 4. Prevalence of comorbidities by age group and demographic details**

| **Age, years** | **Viual disorder, *n* (%)**  **(*n* = 122)** | **Hearing disorder, *n* (%)**  **(*n* = 106)** | **Gait disorder, *n* (%)**  **(*n*=33)** |
| --- | --- | --- | --- |
| 20-24 | 1 (3) | 0 (0) | 1 (3) |
| 25-29 | 2 (3) | 2 (3) | 3 (5) |
| 30-34 | 3 (3) | 2 (2) | 0 (0) |
| 35-39 | 6 (5) | 3 (2) | 0 (0) |
| 40-44 | 7 (5) | 1 (1) | 1 (1) |
| 45-49 | 11 (5) | 5 (2) | 3 (1) |
| 50-54 | 9 (4) | 5 (2) | 2 (2) |
| 55-59 | 13 (7) | 11 (6) | 5 (3) |
| 60-64 | 16 (8) | 13 (7) | 3 (2) |
| 65-69 | 26 (12) | 24 (11) | 8 (4) |
| 70-74 | 16 (10) | 17 (11) | 4 (3) |
| 75-79 | 9 (10) | 8 (9) | 1 (1) |
| 80-84 | 1 (2) | 8 (19) | 2 (5) |
| 85-89 | 0 (0) | 5 (28) | 0 (0) |
| 90-94 | 2 (25) | 2 (25) | 0 (0) |
| 95-99 | 0 (0) | 0 (0) | 0 (0) |

| **Age, years** | **Hypertension, *n* (%)**  **(*n* = 287)** | **Dyslipidemias, *n* (%)**  **(*n* = 219)** | **Diabetes, *n* (%)**  **(*n* = 96)** |
| --- | --- | --- | --- |
| 20-24 | 0 (0) | 2 (6) | 0 (0) |
| 25-29 | 0 (0) | 0 (0) | 2 (3) |
| 30-34 | 2 (2) | 5 (6) | 0 (0) |
| 35-39 | 6 (5) | 7 (6) | 3 (2) |
| 40-44 | 3 (2) | 7 (5) | 3 (2) |
| 45-49 | 15 (6) | 18 (7) | 6 (2) |
| 50-54 | 13 (6) | 13 (6) | 5 (2) |
| 55-59 | 13 (7) | 15 (8) | 5 (3) |
| 60-64 | 31 (16) | 27 (14) | 14 (7) |
| 65-69 | 68 (31) | 46 (21) | 26 (12) |
| 70-74 | 58 (37) | 39 (25) | 13 (8) |
| 75-79 | 43 (46) | 21 (23) | 14 (15) |
| 80-84 | 23 (55) | 11 (26) | 3 (7) |
| 85-89 | 6 (33) | 5 (28) | 0 (0) |
| 90-94 | 6 (75) | 3 (38 | 2 (25) |
| 95-99 | 0 (0) | 0 (0) | 0 (0) |

| **Age, years** | **TBI, *n* (%)**  **(*n* = 41)** | **Stroke, *n* (%)**  **(*n* = 36)** | **Depression, *n* (%)**  **(*n* = 29)** |
| --- | --- | --- | --- |
| 20-24 | 0 (0) | 0 (0) | 0 (0) |
| 25-29 | 2 (3) | 1 (2) | 1 (2) |
| 30-34 | 0 (0) | 0 (0) | 0 (0) |
| 35-39 | 1 (1) | 2 (2) | 1 (1) |
| 40-44 | 2 (1) | 1 (1) | 3 (2) |
| 45-49 | 2 (1) | 3 (1) | 3 (1) |
| 50-54 | 6 (3) | 0 (0) | 3 (1) |
| 55-59 | 6 (3) | 1 (1) | 4 (2) |
| 60-64 | 5 (3) | 6 (3) | 3 (2) |
| 65-69 | 6 (3) | 7 (3) | 8 (4) |
| 70-74 | 4 (3) | 5 (3) | 2 (1) |
| 75-79 | 5 (5) | 3 (3) | 0 (0) |
| 80-84 | 1 (2) | 3 (7) | 1 (2) |
| 85-89 | 1 (6) | 2 (11) | 0 (0) |
| 90-94 | 0 (0) | 2 (25) | 0 (0) |
| 95-99 | 0 (0) | 0 (0) | 0 (0) |

Abbreviation: TBI, Traumatic brain injury.

| **Age, years** | **Epilepsy, *n* (%)**  **(*n* = 782)** |
| --- | --- |
| 20-24 | 13 (36) |
| 25-29 | 26 (40) |
| 30-34 | 41 (46) |
| 35-39 | 63 (52) |
| 40-44 | 71 (52) |
| 45-49 | 126 (52) |
| 50-54 | 111 (53) |
| 55-59 | 91 (46) |
| 60-64 | 90 (46) |
| 65-69 | 78 (35) |
| 70-74 | 45 (28) |
| 75-79 | 16 (17) |
| 80-84 | 9 (21) |
| 85-89 | 2 (11) |
| 90-94 | 0 (0) |
| 95-99 | 0 (0) |
